# Ethyl 4-(2-eth­oxy-2-oxoeth­yl)-3-oxo-4,13-di­aza­penta­cyclo­[11.8.0.0^2,11^.0^5,10^.0^14,19^]henicosa-1,5(10),6,8,11,14(19),15,17,20-nona­ene-12-carboxyl­ate

**DOI:** 10.1107/S1600536813015833

**Published:** 2013-06-22

**Authors:** Qing-Hua Meng, Ya-Nan Wu, Ke Jiang, Yun Liu

**Affiliations:** a School of Chemistry and Chemical Engineering, Jiangsu Normal University, Xuzhou, Jiangsu 221116, People’s Republic of China

## Abstract

In the title compound, C_26_H_22_N_2_O_5_, the system consisting of five fused rings, being essentially planar with an r.m.s. deviation from the least-squares plane of 0.049 (3) Å, makes a dihedral angle of 58.72 (12)° with the plane of the ethyl carboxyl­ate group immediately attached to it, and a dihedral angle of 89.48 (14)° with the plane of the ethyl carboxyl­ate group attached *via* the –CH_2_– bridge. Bond lengths indicate π-delocalization over the whole penta­cyclic system. The mol­ecular conformation is stabilized by a weak intra­molecular C—H⋯O hydrogen bond. In the crystal, mol­ecules form stacks along the *b*-axis direction, neighboring mol­ecules within each stack being related by inversion and the shortest distance between the centroids of the pyridine rings within the stack being 3.667 (2) Å.

## Related literature

For pharmaceutical properties of indolizines and related compounds, see: Olden *et al.* (1991 ▶); Jaffrezou *et al.* (1992 ▶). For the preparation of annulated indolizine, see: Liu *et al.* (2010 ▶). For standard bond-length data, see: Allen *et al.* (1987 ▶).
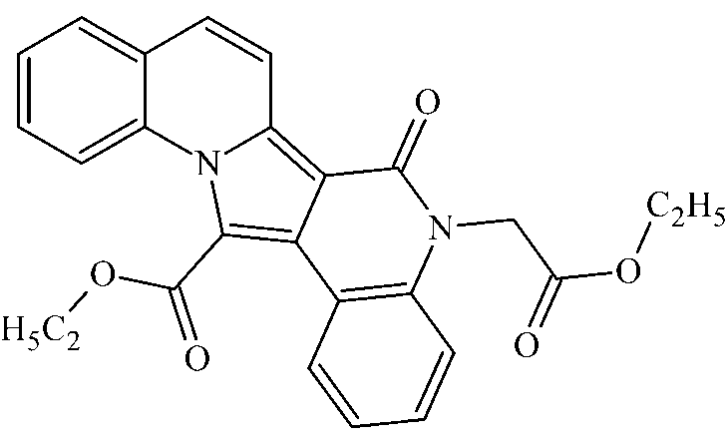


## Experimental

### 

#### Crystal data

C_26_H_22_N_2_O_5_
*M* *_r_* = 442.46
Triclinic, 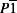

*a* = 8.4000 (17) Å
*b* = 11.008 (2) Å
*c* = 12.304 (3) Å
α = 74.33 (3)°
β = 75.38 (3)°
γ = 86.18 (3)°
*V* = 1060.0 (4) Å^3^
*Z* = 2
Mo *K*α radiation
μ = 0.10 mm^−1^
*T* = 295 K
0.3 × 0.2 × 0.2 mm

#### Data collection

Enraf–Nonius CAD-4 diffractometer
Absorption correction: ψ scan (North *et al.*, 1968 ▶) *T*_min_ = 0.977, *T*_max_ = 0.981
4095 measured reflections
3811 independent reflections
2855 reflections with *I* > 2σ(*I*)
*R*_int_ = 0.021
3 standard reflections every 200 reflections intensity decay: none

#### Refinement

*R*[*F* ^2^ > 2σ(*F* ^2^)] = 0.055
*wR*(*F* ^2^) = 0.153
*S* = 1.01
3809 reflections
301 parameters
H-atom parameters constrained
Δρ_max_ = 0.22 e Å^−3^
Δρ_min_ = −0.25 e Å^−3^

### 

Data collection: *CAD-4 Software* (Enraf–Nonius, 1989 ▶); cell refinement: *CAD-4 Software*; data reduction: *XCAD4* (Harms & Wocadlo, 1995 ▶); program(s) used to solve structure: *SHELXS97* (Sheldrick, 2008 ▶); program(s) used to refine structure: *SHELXL97* (Sheldrick, 2008 ▶); molecular graphics: *SHELXTL* (Sheldrick, 2008 ▶); software used to prepare material for publication: *SHELXTL* and *PLATON* (Spek, 2009 ▶).

## Supplementary Material

Crystal structure: contains datablock(s) I, global. DOI: 10.1107/S1600536813015833/yk2094sup1.cif

Structure factors: contains datablock(s) I. DOI: 10.1107/S1600536813015833/yk2094Isup2.hkl

Supplementary material file. DOI: 10.1107/S1600536813015833/yk2094Isup3.cml

Additional supplementary materials:  crystallographic information; 3D view; checkCIF report

## Figures and Tables

**Table 1:** Hydrogen-bond geometry (Å, °)

*D*—H⋯*A*	*D*—H	H⋯*A*	*D*⋯*A*	*D*—H⋯*A*
C5—H5⋯O4	0.93	2.34	3.168 (3)	148

**Fig. 1.**
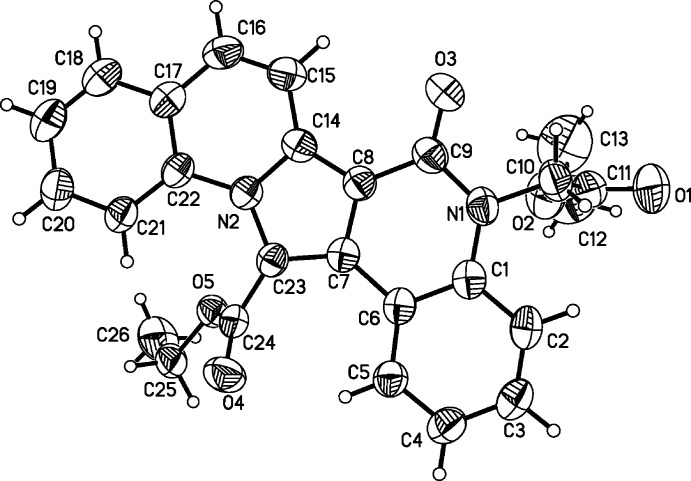
The molecular structure of the title molecule, showing the atom-labelling scheme. Displacement ellipsoids are drawn at the 50% probability level.

**Fig. 2.**
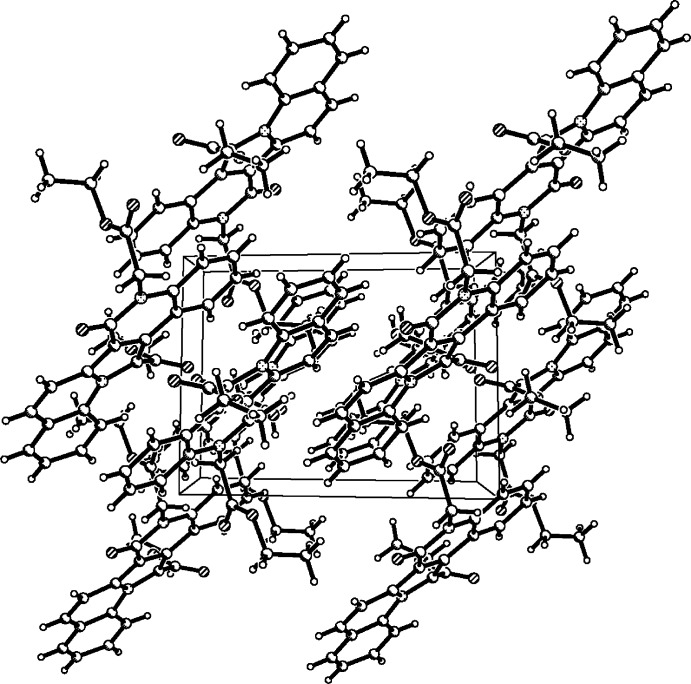
Packing diagram of the title compounds, viewed along the crystallographic c axis.

## supplementary crystallographic information

### Comment

The natural and many synthetic indolizines show a diversity of biological activity and are playing an increasingly important role in developing of new pharmaceuticals (Olden *et al.*, 1991; Jaffrezou *et al.*, 1992). The synthesis of these compounds has drawn much research interest (Liu *et al.*, 2010). Indolizino[1,2-*c*]quinolin-6(5*H*)-one is an important annulated indolizines derivative. In our ongoing research work on the direct one pot syntheses of this class of compounds, we have prepared the title compound, (I), as one of the products. As part of this study, we have undertaken an X-ray crystallographic analysis of (I) in order to confirm its structure further.

The bond lengths and angles of the title molecule (Fig. 1) are within normal ranges (Allen *et al.*, 1987). The two quinoline rings containing N1 [r.m.s. deviation from mean plane of 0.0131 (3) Å] and N2 [r.m.s. deviation = 0.0573 Å] are make dihedral angles with the pyrrole ring of 4.16 (12)° and 6.30 (13)°. The molecular conformation is stabilized by weak intramolecular C—H···O hydrogen bonds (Table 1). The packing of the title molecules *via* the π -π stacking interaction is shown in Fig.2, with a shortest intercentroid distance of 3.627 (2) Å.

### Experimental

The compound (I) was prepared by the reaction of 1-(2-ethoxy-2-oxoethyl)quinolinium salt (2.0 mmol), tetrakispyridinecobalt(II) di(hydrochromate) [CoPy_4_](HCrO_4_)_2_ (1.0 g) and potassium carbonate (3.0 mmol) mixed in 10 mL CH_3_CN and heated at reflux for 4 h. After the reaction was completed, the reaction mixture was purified by column chromatography on silica gel, and the product was isolated after evaporation of the solvent. Single crystals of (I) were obtained by slow evaporation from a petroleum ether–ethyl acetate (3:1) solvent system (yield 52%).

### Refinement

The H atoms were geometrically placed and were treated as riding, with C—H = 0.93 Å.

### Crystal data

**Table:**

C_26_H_22_N_2_O_5_	*Z* = 2
*M**_r_* = 442.46	*F*(000) = 464
Triclinic, *P*1	*D*_x_ = 1.386 Mg m^−^^3^
Hall symbol: -P 1	Mo *K*α radiation, λ = 0.71073 Å
*a* = 8.4000 (17) Å	Cell parameters from 25 reflections
*b* = 11.008 (2) Å	θ = 9–12°
*c* = 12.304 (3) Å	µ = 0.10 mm^−^^1^
α = 74.33 (3)°	*T* = 295 K
β = 75.38 (3)°	Block, colourless
γ = 86.18 (3)°	0.3 × 0.2 × 0.2 mm
*V* = 1060.0 (4) Å^3^	

### Data collection

**Table:**

Enraf–Nonius CAD-4 diffractometer	2855 reflections with *I* > 2σ(*I*)
Radiation source: fine-focus sealed tube	*R*_int_ = 0.021
Graphite monochromator	θ_max_ = 25.2°, θ_min_ = 1.8°
ω/2θ scans	*h* = 0→10
Absorption correction: ψ scan (North *et al.*, 1968)	*k* = −13→13
*T*_min_ = 0.977, *T*_max_ = 0.981	*l* = −14→14
4095 measured reflections	3 standard reflections every 200 reflections
3811 independent reflections	intensity decay: none

### Refinement

**Table:**

Refinement on *F*^2^	Secondary atom site location: difference Fourier map
Least-squares matrix: full	Hydrogen site location: inferred from neighbouring sites
*R*[*F*^2^ > 2σ(*F*^2^)] = 0.055	H-atom parameters constrained
*wR*(*F*^2^) = 0.153	*w* = 1/[σ^2^(*F*_o_^2^) + (0.067*P*)^2^ + 0.6558*P*] where *P* = (*F*_o_^2^ + 2*F*_c_^2^)/3
*S* = 1.01	(Δ/σ)_max_ < 0.001
3809 reflections	Δρ_max_ = 0.22 e Å^−^^3^
301 parameters	Δρ_min_ = −0.25 e Å^−^^3^
0 restraints	Extinction correction: *SHELXL97* (Sheldrick, 2008), Fc^*^=kFc[1+0.001xFc^2^λ^3^/sin(2θ)]^-1/4^
Primary atom site location: structure-invariant direct methods	Extinction coefficient: 0.056 (4)

### Special details

**Table:**

Geometry. All s.u.'s (except the s.u. in the dihedral angle between two l.s. planes) are estimated using the full covariance matrix. The cell s.u.'s are taken into account individually in the estimation of s.u.'s in distances, angles and torsion angles; correlations between s.u.'s in cell parameters are only used when they are defined by crystal symmetry. An approximate (isotropic) treatment of cell s.u.'s is used for estimating s.u.'s involving l.s. planes.
Refinement. Refinement of *F*^2^ against ALL reflections. The weighted *R*-factor *wR* and goodness of fit *S* are based on *F*^2^, conventional *R*-factors *R* are based on *F*, with *F* set to zero for negative *F*^2^. The threshold expression of *F*^2^ > σ(*F*^2^) is used only for calculating *R*-factors(gt) *etc*. and is not relevant to the choice of reflections for refinement. *R*-factors based on *F*^2^ are statistically about twice as large as those based on *F*, and *R*- factors based on ALL data will be even larger.Two reflections were omitted from the initial data set of 3811 reflections, thus 3809 is the correct number of reflections in the L.S. refinement procedure

### Fractional atomic coordinates and isotropic or equivalent isotropic displacement parameters (Å^2^)

**Table:**

	*x*	*y*	*z*	*U*_iso_*/*U*_eq_	
O5	0.3913 (2)	0.80663 (16)	0.26370 (14)	0.0453 (4)	
N2	0.5328 (2)	0.73427 (18)	0.46629 (17)	0.0413 (5)	
O3	0.3219 (3)	0.7099 (2)	0.82722 (16)	0.0659 (6)	
C7	0.3563 (3)	0.8813 (2)	0.5236 (2)	0.0394 (5)	
N1	0.1884 (2)	0.8865 (2)	0.75042 (17)	0.0475 (5)	
C8	0.3822 (3)	0.7900 (2)	0.6224 (2)	0.0428 (6)	
O4	0.4712 (3)	1.00189 (17)	0.24670 (17)	0.0650 (6)	
C6	0.2412 (3)	0.9830 (2)	0.5397 (2)	0.0411 (6)	
C1	0.1577 (3)	0.9812 (2)	0.6552 (2)	0.0437 (6)	
C14	0.4914 (3)	0.7000 (2)	0.5869 (2)	0.0437 (6)	
C24	0.4415 (3)	0.8959 (2)	0.3036 (2)	0.0426 (6)	
C5	0.2067 (3)	1.0811 (2)	0.4496 (2)	0.0474 (6)	
H5	0.2624	1.0842	0.3733	0.057*	
C23	0.4492 (3)	0.8453 (2)	0.4264 (2)	0.0404 (5)	
C21	0.7233 (3)	0.7114 (3)	0.2844 (2)	0.0494 (6)	
H21	0.6902	0.7884	0.2427	0.059*	
C2	0.0422 (3)	1.0757 (3)	0.6742 (2)	0.0521 (7)	
H2	−0.0142	1.0748	0.7499	0.062*	
C22	0.6559 (3)	0.6685 (2)	0.4033 (2)	0.0429 (6)	
C9	0.2995 (3)	0.7893 (3)	0.7406 (2)	0.0479 (6)	
C17	0.7146 (3)	0.5562 (2)	0.4673 (2)	0.0530 (7)	
O2	−0.1099 (3)	0.7778 (2)	0.8434 (2)	0.0799 (7)	
C4	0.0929 (3)	1.1733 (3)	0.4702 (3)	0.0544 (7)	
H4	0.0716	1.2373	0.4087	0.065*	
C15	0.5548 (4)	0.5884 (3)	0.6489 (2)	0.0541 (7)	
H15	0.5243	0.5646	0.7298	0.065*	
C3	0.0109 (3)	1.1694 (3)	0.5831 (3)	0.0552 (7)	
H3	−0.0665	1.2311	0.5977	0.066*	
O1	−0.1713 (3)	0.8786 (3)	0.9833 (2)	0.0916 (8)	
C25	0.3949 (4)	0.8401 (3)	0.1403 (2)	0.0573 (7)	
H25A	0.5061	0.8612	0.0938	0.069*	
H25B	0.3247	0.9123	0.1213	0.069*	
C10	0.1035 (4)	0.8897 (3)	0.8679 (2)	0.0616 (8)	
H10A	0.1092	0.9750	0.8748	0.074*	
H10B	0.1614	0.8352	0.9215	0.074*	
C11	−0.0746 (4)	0.8496 (3)	0.9045 (2)	0.0618 (8)	
C16	0.6595 (4)	0.5167 (3)	0.5907 (3)	0.0590 (7)	
H16	0.6962	0.4405	0.6314	0.071*	
C20	0.8391 (4)	0.6400 (3)	0.2282 (3)	0.0652 (8)	
H20	0.8845	0.6694	0.1486	0.078*	
C19	0.8888 (4)	0.5240 (3)	0.2896 (3)	0.0799 (10)	
H19	0.9619	0.4736	0.2506	0.096*	
C13	−0.2873 (6)	0.6002 (5)	0.9349 (5)	0.136 (2)	
H13A	−0.2511	0.5893	1.0050	0.205*	
H13B	−0.2182	0.5518	0.8871	0.205*	
H13C	−0.3990	0.5717	0.9543	0.205*	
C18	0.8299 (4)	0.4852 (3)	0.4063 (3)	0.0709 (9)	
H18	0.8670	0.4093	0.4472	0.085*	
C26	0.3346 (5)	0.7286 (4)	0.1163 (3)	0.0830 (11)	
H26A	0.2217	0.7128	0.1580	0.124*	
H26B	0.3998	0.6563	0.1410	0.124*	
H26C	0.3433	0.7446	0.0343	0.124*	
C12	−0.2776 (4)	0.7282 (4)	0.8740 (4)	0.1070 (15)	
H12A	−0.3505	0.7763	0.9216	0.128*	
H12B	−0.3147	0.7388	0.8035	0.128*	

### Atomic displacement parameters (Å^2^)

**Table:**

	*U*^11^	*U*^22^	*U*^33^	*U*^12^	*U*^13^	*U*^23^
O5	0.0468 (10)	0.0508 (10)	0.0396 (9)	0.0018 (8)	−0.0118 (7)	−0.0134 (8)
N2	0.0410 (11)	0.0398 (11)	0.0445 (11)	0.0001 (9)	−0.0108 (9)	−0.0132 (9)
O3	0.0785 (14)	0.0722 (13)	0.0418 (11)	0.0085 (11)	−0.0132 (10)	−0.0097 (10)
C7	0.0340 (12)	0.0429 (13)	0.0439 (13)	−0.0032 (10)	−0.0103 (10)	−0.0142 (11)
N1	0.0395 (11)	0.0613 (14)	0.0418 (12)	0.0005 (10)	−0.0062 (9)	−0.0173 (10)
C8	0.0387 (13)	0.0470 (14)	0.0429 (13)	−0.0009 (11)	−0.0099 (10)	−0.0119 (11)
O4	0.0882 (15)	0.0437 (11)	0.0535 (11)	−0.0043 (10)	−0.0101 (10)	−0.0026 (9)
C6	0.0326 (12)	0.0465 (14)	0.0464 (14)	−0.0041 (10)	−0.0080 (10)	−0.0165 (11)
C1	0.0340 (12)	0.0522 (15)	0.0484 (14)	−0.0060 (11)	−0.0090 (11)	−0.0184 (12)
C14	0.0448 (14)	0.0439 (14)	0.0421 (13)	−0.0044 (11)	−0.0119 (11)	−0.0085 (11)
C24	0.0364 (13)	0.0450 (14)	0.0431 (13)	0.0043 (11)	−0.0044 (10)	−0.0117 (11)
C5	0.0417 (14)	0.0495 (15)	0.0492 (15)	0.0006 (11)	−0.0089 (11)	−0.0124 (12)
C23	0.0356 (12)	0.0406 (13)	0.0452 (13)	−0.0011 (10)	−0.0081 (10)	−0.0127 (10)
C21	0.0400 (14)	0.0538 (15)	0.0537 (16)	0.0064 (12)	−0.0098 (12)	−0.0159 (12)
C2	0.0418 (14)	0.0595 (17)	0.0559 (16)	−0.0022 (12)	−0.0016 (12)	−0.0258 (14)
C22	0.0354 (12)	0.0436 (13)	0.0528 (15)	0.0021 (10)	−0.0119 (11)	−0.0171 (11)
C9	0.0486 (15)	0.0540 (15)	0.0422 (14)	−0.0035 (12)	−0.0124 (12)	−0.0121 (12)
C17	0.0508 (16)	0.0495 (15)	0.0601 (17)	0.0073 (12)	−0.0148 (13)	−0.0172 (13)
O2	0.0557 (13)	0.0863 (16)	0.0884 (16)	−0.0132 (11)	0.0051 (11)	−0.0250 (13)
C4	0.0478 (15)	0.0480 (15)	0.0651 (18)	0.0049 (12)	−0.0133 (13)	−0.0126 (13)
C15	0.0614 (17)	0.0506 (15)	0.0490 (15)	0.0015 (13)	−0.0179 (13)	−0.0072 (12)
C3	0.0437 (15)	0.0514 (16)	0.0715 (19)	0.0057 (12)	−0.0091 (13)	−0.0237 (14)
O1	0.0767 (16)	0.1089 (19)	0.0682 (15)	0.0147 (14)	0.0162 (12)	−0.0232 (14)
C25	0.0567 (17)	0.0748 (19)	0.0394 (14)	0.0133 (14)	−0.0130 (12)	−0.0154 (13)
C10	0.0599 (18)	0.083 (2)	0.0438 (15)	0.0119 (15)	−0.0113 (13)	−0.0247 (14)
C11	0.0578 (18)	0.0673 (19)	0.0451 (16)	0.0095 (15)	0.0018 (14)	−0.0050 (14)
C16	0.0662 (18)	0.0453 (15)	0.0630 (18)	0.0106 (13)	−0.0211 (15)	−0.0076 (13)
C20	0.0519 (17)	0.080 (2)	0.0603 (18)	0.0102 (15)	−0.0039 (14)	−0.0240 (16)
C19	0.074 (2)	0.080 (2)	0.080 (2)	0.0343 (18)	−0.0092 (18)	−0.0302 (19)
C13	0.114 (4)	0.101 (4)	0.178 (5)	−0.043 (3)	0.000 (4)	−0.031 (3)
C18	0.073 (2)	0.0600 (18)	0.075 (2)	0.0256 (16)	−0.0161 (17)	−0.0185 (16)
C26	0.093 (3)	0.113 (3)	0.0584 (19)	−0.004 (2)	−0.0301 (18)	−0.037 (2)
C12	0.055 (2)	0.100 (3)	0.136 (4)	−0.023 (2)	0.001 (2)	0.001 (3)

### Geometric parameters (Å, º)

**Table:**

O5—C24	1.344 (3)	O2—C11	1.320 (4)
O5—C25	1.456 (3)	O2—C12	1.466 (4)
N2—C14	1.385 (3)	C4—C3	1.376 (4)
N2—C23	1.401 (3)	C4—H4	0.9300
N2—C22	1.412 (3)	C15—C16	1.344 (4)
O3—C9	1.229 (3)	C15—H15	0.9300
C7—C23	1.392 (3)	C3—H3	0.9300
C7—C8	1.406 (3)	O1—C11	1.199 (4)
C7—C6	1.452 (3)	C25—C26	1.484 (4)
N1—C9	1.383 (3)	C25—H25A	0.9700
N1—C1	1.408 (3)	C25—H25B	0.9700
N1—C10	1.450 (3)	C10—C11	1.509 (4)
C8—C14	1.387 (3)	C10—H10A	0.9700
C8—C9	1.443 (3)	C10—H10B	0.9700
O4—C24	1.192 (3)	C16—H16	0.9300
C6—C5	1.397 (3)	C20—C19	1.394 (5)
C6—C1	1.413 (3)	C20—H20	0.9300
C1—C2	1.400 (4)	C19—C18	1.352 (5)
C14—C15	1.409 (4)	C19—H19	0.9300
C24—C23	1.479 (3)	C13—C12	1.403 (6)
C5—C4	1.377 (4)	C13—H13A	0.9600
C5—H5	0.9300	C13—H13B	0.9600
C21—C20	1.376 (4)	C13—H13C	0.9600
C21—C22	1.388 (4)	C18—H18	0.9300
C21—H21	0.9300	C26—H26A	0.9600
C2—C3	1.371 (4)	C26—H26B	0.9600
C2—H2	0.9300	C26—H26C	0.9600
C22—C17	1.407 (4)	C12—H12A	0.9700
C17—C18	1.400 (4)	C12—H12B	0.9700
C17—C16	1.423 (4)		
			
C24—O5—C25	116.4 (2)	C16—C15—H15	120.1
C14—N2—C23	109.1 (2)	C14—C15—H15	120.1
C14—N2—C22	120.8 (2)	C2—C3—C4	120.8 (3)
C23—N2—C22	129.7 (2)	C2—C3—H3	119.6
C23—C7—C8	107.1 (2)	C4—C3—H3	119.6
C23—C7—C6	133.9 (2)	O5—C25—C26	107.0 (2)
C8—C7—C6	118.8 (2)	O5—C25—H25A	110.3
C9—N1—C1	124.3 (2)	C26—C25—H25A	110.3
C9—N1—C10	116.1 (2)	O5—C25—H25B	110.3
C1—N1—C10	119.6 (2)	C26—C25—H25B	110.3
C14—C8—C7	109.1 (2)	H25A—C25—H25B	108.6
C14—C8—C9	126.6 (2)	N1—C10—C11	115.0 (2)
C7—C8—C9	124.2 (2)	N1—C10—H10A	108.5
C5—C6—C1	118.2 (2)	C11—C10—H10A	108.5
C5—C6—C7	124.8 (2)	N1—C10—H10B	108.5
C1—C6—C7	117.1 (2)	C11—C10—H10B	108.5
C2—C1—N1	119.9 (2)	H10A—C10—H10B	107.5
C2—C1—C6	118.8 (2)	O1—C11—O2	124.4 (3)
N1—C1—C6	121.2 (2)	O1—C11—C10	122.3 (3)
N2—C14—C8	107.1 (2)	O2—C11—C10	113.2 (2)
N2—C14—C15	120.4 (2)	C15—C16—C17	120.5 (3)
C8—C14—C15	132.5 (2)	C15—C16—H16	119.7
O4—C24—O5	123.4 (2)	C17—C16—H16	119.7
O4—C24—C23	125.8 (2)	C21—C20—C19	120.5 (3)
O5—C24—C23	110.8 (2)	C21—C20—H20	119.8
C4—C5—C6	122.1 (2)	C19—C20—H20	119.8
C4—C5—H5	119.0	C18—C19—C20	119.5 (3)
C6—C5—H5	119.0	C18—C19—H19	120.2
C7—C23—N2	107.6 (2)	C20—C19—H19	120.2
C7—C23—C24	128.1 (2)	C12—C13—H13A	109.5
N2—C23—C24	123.4 (2)	C12—C13—H13B	109.5
C20—C21—C22	120.0 (3)	H13A—C13—H13B	109.5
C20—C21—H21	120.0	C12—C13—H13C	109.5
C22—C21—H21	120.0	H13A—C13—H13C	109.5
C3—C2—C1	121.1 (2)	H13B—C13—H13C	109.5
C3—C2—H2	119.5	C19—C18—C17	121.7 (3)
C1—C2—H2	119.5	C19—C18—H18	119.1
C21—C22—C17	119.7 (2)	C17—C18—H18	119.1
C21—C22—N2	123.3 (2)	C25—C26—H26A	109.5
C17—C22—N2	116.9 (2)	C25—C26—H26B	109.5
O3—C9—N1	121.2 (2)	H26A—C26—H26B	109.5
O3—C9—C8	124.5 (3)	C25—C26—H26C	109.5
N1—C9—C8	114.3 (2)	H26A—C26—H26C	109.5
C18—C17—C22	118.2 (3)	H26B—C26—H26C	109.5
C18—C17—C16	120.9 (3)	C13—C12—O2	112.1 (4)
C22—C17—C16	120.9 (2)	C13—C12—H12A	109.2
C11—O2—C12	118.4 (3)	O2—C12—H12A	109.2
C5—C4—C3	119.1 (3)	C13—C12—H12B	109.2
C5—C4—H4	120.4	O2—C12—H12B	109.2
C3—C4—H4	120.4	H12A—C12—H12B	107.9
C16—C15—C14	119.7 (3)		

### Hydrogen-bond geometry (Å, º)

**Table:**

*D*—H···*A*	*D*—H	H···*A*	*D*···*A*	*D*—H···*A*
C12—H12*A*···O1	0.97	2.32	2.712 (5)	103
C10—H10*B*···O3	0.97	2.21	2.669 (4)	107
C15—H15···O3	0.93	2.56	3.082 (4)	116
C21—H21···O5	0.93	2.46	2.964 (3)	114
C5—H5···O4	0.93	2.34	3.168 (3)	148

## References

[bb1] Allen, F. H., Kennard, O., Watson, D. G., Brammer, L., Orpen, A. G. & Taylor, R. (1987). *J. Chem. Soc. Perkin Trans. 2*, pp. S1–19.

[bb2] Enraf–Nonius (1989). *CAD-4 Software* Enraf–Nonius, Delft, The Netherlands.

[bb3] Harms, K. & Wocadlo, S. (1995). *XCAD4* University of Marburg, Germany.

[bb4] Jaffrezou, J. P., Levade, T., Thurneyssen, O., Chiron, M., Bordier, C., Attal, M., Chatelain, P. & Laurent, G. (1992). *Cancer Res.* **52**, 1352–1359. 1737397

[bb5] Liu, Y., Hu, H.-Y., Zhang, Y., Hu, H.-W. & Xu, J.-H. (2010). *Org. Biomol. Chem.* **8**, 4921–4926. 10.1039/c0ob00299b 20740245

[bb6] North, A. C. T., Phillips, D. C. & Mathews, F. S. (1968). *Acta Cryst.* A**24**, 351–359.

[bb7] Olden, K., Breton, P., Grzegorzevski, K., Yasuda, Y., Gause, B. L., Oredipe, O. A., Newton, S. A. & White, S. L. (1991). *Pharmacol. Ther.* **50**, 285–290. 10.1016/0163-7258(91)90046-o 1754603

[bb8] Sheldrick, G. M. (2008). *Acta Cryst.* A**64**, 112–122. 10.1107/S0108767307043930 18156677

[bb9] Spek, A. L. (2009). *Acta Cryst.* D**65**, 148–155. 10.1107/S090744490804362X PMC2631630 19171970

